# Design and evaluation of ^32^P-labeled hydroxyapatite nanoparticles for bone tumor therapy

**DOI:** 10.1080/10717544.2023.2168791

**Published:** 2023-01-23

**Authors:** Dongliang Zhai, Yumei Wang, Songke Yu, Jiren Zhou, Jia Song, Shilei Hao, Xiaoliang Chen

**Affiliations:** a Department of Nuclear Medicine, Chongqing University Cancer Hospital, Chongqing, China; b Key Laboratory of Biorheological Science and Technology, Ministry of Education, College of Bioengineering, Chongqing University, Chongqing, China; c Chongqing Key Laboratory of Translational Research for Cancer Metastasis and Individualized Treatment, Chongqing University Cancer Hospital and Chongqing Cancer Institute and Chongqing Cancer Hospital, Chongqing, China

**Keywords:** Cancer, bone treating, hydroxyapatite, ^32^P, chemical synthesis

## Abstract

The clinical diagnosis and treatment of malignant bone tumors are still major clinical challenges due to their high incidence are difficulty. Targeted therapies have become a critical approach to treat bone tumors. In recent years, radiopharmaceuticals have been used widely and have shown potent and efficient results in treating bone tumors, among which ^32^P and the labeled radiopharmaceuticals play an essential role. In this study, the ^32^P-labeled hydroxyapatite (HA) was prepared through chemical synthesis (^32^P-Hap) and physical adsorption (^32^P-doped-Hap). The in vitro stability of ^32^P-labeled HA was analyzed to assess the superiority of the new-found chemical synthesis. The radiolabeling yield and stability of chemical synthesis (97.6 ± 0.5%) were significantly improved compared with physical adsorption (92.7 ± 0.4%). Furthermore, the CT results corroborate that ^32^P-Hap (100 μCi) +DOX group has the highest tumor suppression rate and can effectively reduce bone destruction. The results corroborate the effectiveness of the chemical synthesis and validate the application of ^32^P-Hap in bone tumors. Therefore, ^32^P-Hap (100 μCi) + DOX may be an effective strategy for bone metastasis treatments.

## 1. Introduction

In recent studies, radiolabeled particulate materials have been widely applied to solid tumor treatment. Radionuclides with short-range and high-energy emissions, such as ^32^P (T_½_ = 14.3 d), ^90^Y (T_½_ = 64.1 h), and ^131^I (T_½_ = 8.04 d), are preferred to accurately target into organs of the main β-emitting tumor (Vimalnath et al., 2015; Rial et al., 2021). However, compared with the most medical radionuclides, ^32^P has a long half-life, and the activity declines slowly than them over time, which can be maintained a longer time for the therapy of tumor. In addition, since ^32^P has been adopted in more and more radiotherapy cases with a longer half-time and more practical performance than ^90^Y and ^131^I. Its safety and efficacy in tumor treatment have already been reported previously (Rajeswari et al., 2016; Yang et al., 2020).

HA, a well-performed nanomaterial, has been widely used in dental and biomedical fields for its great biocompatibility and bioactivity (Sun et al., 2019; Rial et al., 2021; De Lama-Odria et al., 2022). The HA is chemically defined as Ca_10_(PO_4_)_6_(OH)_2_ with a constant Ca/P ratio. In addition, P is a constituent element of hydroxyapatite, once incorporated into the molecular framework, it is highly unlikely that ^32^P activity would leach out from ^32^P-labeled hydroxyapatite particles in vivo. Thus, ^32^P-labeled HA will be a potential therapeutic strategy for bone tumors. Among the various synthetic methods, a straightforward aqueous precipitation route stands out by using CaCl_2_ and Na_3_PO_4_ as starting materials. ^32^P-labeled HA particles can be obtained by mixing Na_2_H[^32^P]PO_4_ with analytically pure Na_3_PO_4_ or NaH_2_PO_4_. This method immensely simplifies the preparation of ^32^P-labeled HA and maintains high radiochemical purity and favorable stability compared with previous reports. The HA has shown an inhibitory effect on various cancer cells, including breast cancer, liver cancer, gastric cancer, and carcinosarcoma cells (De Lama-Odria et al., 2022; Kang et al., 2022; Xu et al., 2022). Excess calcium uptake will cause toxicity in tumor cells, thus inhibiting their growth. In addition, HA is an excellent drug carrier with many advantages (Liu et al., 2018; Safi et al., 2018; Ying Liu et al., 2019; Liu et al., 2021). Firstly, its great biocompatibility can avoid adverse reactions between materials and body tissue or blood system (Zhang et al., 2017; Banerjee et al., 2018; Kavasi et al., 2021). Secondly, HA is stable under physiological conditions and has strong adsorption property for its large surface area, which ensures a long cycle time of drugs in body (Pandi & Viswanathan, 2015). Finally, appropriate biodegradability ensures that degraded products and drugs released from the core can be absorbed or naturally excluded from the body to exert a therapeutic effect.

This study aimed to synthesize ^32^P-labeled HA for targeted therapy of bone tumors. The ^32^P-labeled HA was prepared through chemical synthesis and physical adsorption. The in vitro stability of ^32^P-labeled HA was analyzed to assess the superiority of the new-found chemical synthesis. Furthermore, the orthotopic bone tumor-bearing mice model was established to investigate if the developed material could effectively treat bone tumors.

## 2. Materials and methods

### 2.1. Materials

Sodium dihydrogen phosphate (NaH_2_PO_4_), Calcium chloride (CaCl_2_), Sodium hydroxide (NaOH), Hydroxyapatite (HA), Potassium bromide (KBr), Sodium iodide (NaI), Saline, Na_2_H[^32^P]PO_4_, and NaH_2_PO_4_ were purchased from Chengdu Cologne Chemical Reagent Factory (China), Doxorubicin (DOX) were purchased from Aladdin Industrial Co. (Shanghai, China), 1% penicillin-streptomycin and 10% (v/v) fetal bovine serum were purchased from HyClone (Waltham, MA, USA). All other materials and reagents used in the study were of analytical grades.

### 2.2. Synthesis and characterization of HA

#### 2.2.1. Synthesis

The preparation of acicular HA is as follows. NaH_2_PO_4_ solution (0.3 mol/L) was dropped into the same volume of CaCl_2_ (0.5 mol/L) at 70 °C and stirred at 600 rpm/min. After dripping, the reaction was stirred for another 2 h. During this procedure, the pH value was ensured at 10 using NaOH solution (1 mol/L), and the stoichiometric Ca/P ratio was maintained at 1.67. The mixture was vortexed thoroughly, followed by centrifugation at 1000 rpm for 5 min. After the supernatant was removed, the precipitate was washed 3–4 times with distilled water through vortexing and centrifuging. The HA powder was obtained after freeze-drying.

#### 2.2.2. Morphological observation

The morphology of the acicular HA was inspected using a scanning electron microscope (SEM, EVOLS 25, FEI, USA) at an accelerating voltage of 5.0 kV. The samples were coated with gold for 15 s before imaging.

#### 2.2.3. Fourier transform-infrared (FT-IR) analysis

The chemical structure of the acicular HA was characterized by an FT-IR spectrometer (Nicolet 550, USA). The HA was mixed with potassium bromide (KBr) and compressed to a thin pellet for examination with a wavenumber range of 400–4000 cm^−1^.

### 2.3. Synthesis of ^32^P-labeled HA

The ^32^P-labeled HA was prepared by two different routes. In two cases, the Ca/P ratio was both maintained at 1.67. The chemical synthesis is to mix NaH_2_PO_4_ (0.3 mol/L) solution with Na_2_H[^32^P]PO_4_ solution (5–6 mCi ^32^P) to replace NaH_2_PO_4_, and the other steps are the same as the synthesis of HA above. The physical adsorption is accomplished by mixing the prepared HA microparticles and Na_2_H[^32^P]PO_4_ (0.2 mL, 5–6 mCi ^32^P) in 1 mL saline. Then, the reaction mixture was stirred at 70 °C for 2 h. The obtained ^32^P-labeled particulates were washed three times with normal saline (1 mL) to remove the free ^32^P activity. Then, the radiolabeling yield and radiochemical purity of the obtained particulates were determined subsequently for further biological studies.

### 2.4. Determination of radiolabeling yield and radiochemical purity

The radiolabeling yield was measured before washing off the free ^32^P activity after the completion of the reaction. Firstly, the reaction mixture was vortexed thoroughly, followed by centrifugation at 1000 rpm for 5 min to stratify. Subsequently, ^32^P activity of half volume of the supernatant and the remaining supernatant solution together with HA particles were measured by NaI (Tl) detector. After counting, the radiolabeling yield was calculated using the following equation:

% Radiolabeling yield = [(Y−X)/(Y+X)] ×100%

X: ^32^P activity of the half volume of the supernatant solution (after decay-corrected).

Y: ^32^P activity of the remaining supernatant solution and HA particles (after decay-corrected).

The radiochemical purity of the ^32^P-labeled HA was determined with the same method after the free ^32^P activity was removed completely by washing.

### 2.5. In vitro stability of ^32^P-labeled HA

The in vitro stability of ^32^P-labeled HA (^32^P-Hap and ^32^P-doped-Hap) microparticles was investigated in 0.9% (w/v) saline to measure the leached activity of ^32^P from the radiolabeled microparticles. ^32^P-HA microparticles (∼1 mCi) suspended in normal saline were added to 2 mL normal saline at different pHs of 5.0, 6.8, and 7.2 and stored at room temperature. The radiochemical purity of ^32^P-labeled HA obtained by two methods was repeatedly determined at the end of the time intervals of day 1, day 2, day 4, day 7, and day 14.

### 2.6. Animal experiments

#### 2.6.1. Orthotopic bone metastasis model

The orthotopic bone tumor-bearing mice model was established to confirm the antitumor efficacy of ^32^P-labeled HA. The guidelines for animal experiments were followed in accordance with the Institutional Animal Care and Use Committee of Chongqing University Cancer Hospital, China. Balb/c mice (female, 6–8 weeks old) purchased from Spitford Biotechnology Co., Ltd, were raised at room temperature (22–25 °C, light/dark cycle) with free access to food and water. Human breast cancer cell line 4 T1 used for establishing bone model was grown in RPMI 1640 medium supplemented with 1% penicillin-streptomycin and 10% (v/v) fetal bovine serum supplemented at 37 °C in an atmosphere of 5% CO_2_, which was provided by Chinese Academy of Sciences Cell Bank.

To establish an orthotopic bone tumor-bearing mice model, 6 weeks Balb/c mice were inoculated in their cavum medulla with 4 T1 cells (10^4^ cells/10 μL) suspended in 50% (v/v) Matrigel. Their body weight and tumor volume were monitored every other day. The volume of the tumors was calculated according to the following equation:

$$V\;=\;\pi /6\times \text{length}\times {\text{width}}^{2}$$

#### 2.6.2. Antitumor efficacy evaluation

The tumor-bearing mice were randomly assigned to 7 groups for different treatments when the volume of tumors reached approximately 200 mm^3^. Saline, doxorubicin (DOX), and different doses of ^32^P-labeled HA microparticles, including ^32^P-doped-Hap (100 μCi), ^32^P-Hap (50 μCi), ^32^P-Hap (100 μCi) ^32^P-Hap (150 μCi), and ^32^P-Hap (100 μCi) +DOX, were injected into the site of the tumor by two methods. The treatment was repeated every 3 days for 3 weeks, and tumor volume and body weight were measured and recorded every other day. The mice were sacrificed with overdose of isoflurane 1 day after the last injection, and tumor tissues were weighed and photographed. The tumor burden was calculated as follows:

$$\text{Tumor Inhibition \% }=\;\frac{w_{0}-w_{1}}{w_{0}}\times 100\%$$

W0: The weight of the tumor in the control group.

W1: The weight of the tumor in each treatment group.

## 3. Results and discussion

### 3.1. Characterization of HA

**Figure 1.**
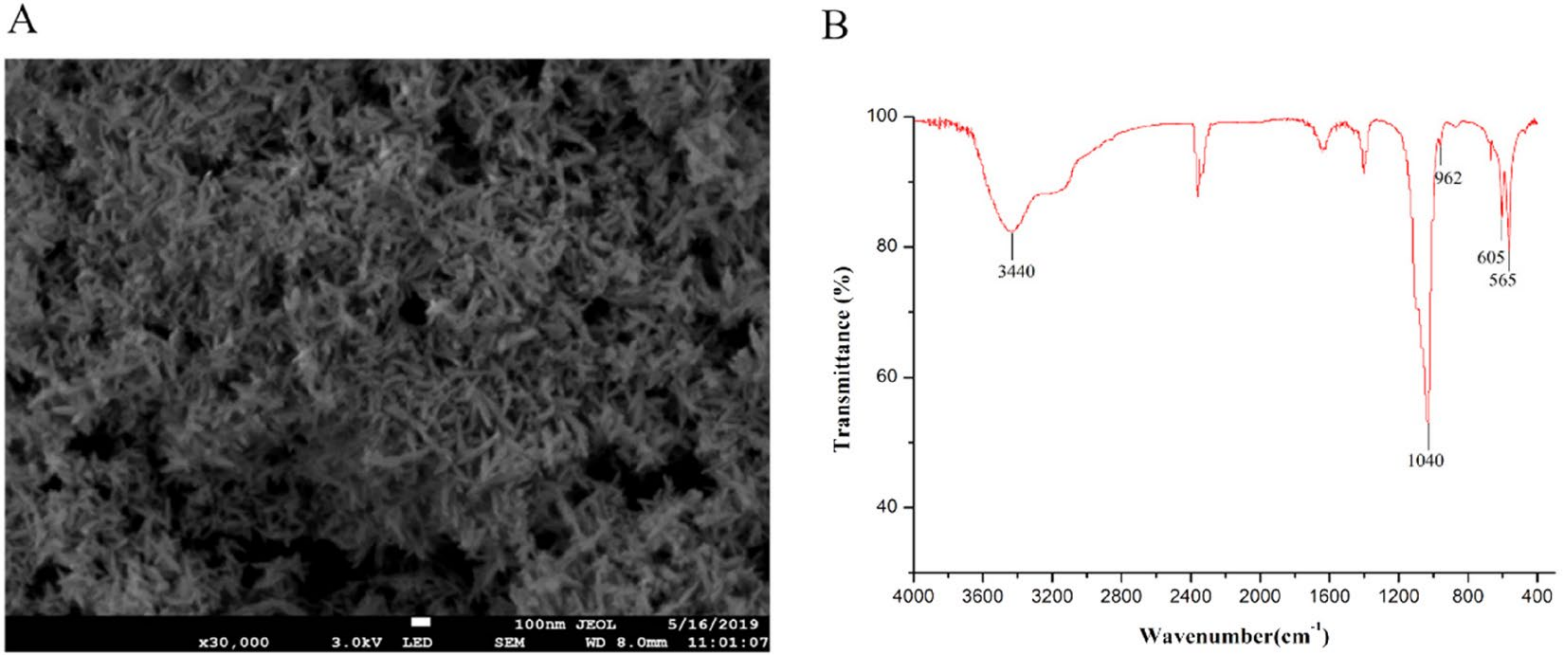
SEM images of HA(A) and the FT-IR spectra of HA (B).

In this study, NaH_2_PO_4_ and CaCl_2_ were used as phosphorus and calcium sources to synthesize HA with a Ca/P ratio of 1.67. The morphology and microstructure of HA were observed on an SEM. As shown in Figure 1(A), the achieved particles are rod-shaped. The FT-IR spectra of HA are displayed in Figure 1(B). The characteristic absorption peak observed at 565, 605, and 1040 cm^−1^ corresponds to the bending and stretching vibration of PO_4_^3-^ of HA. The lattice OH^-^ group has bending deformation at 3440 and 605 cm^−1^. The FT-IR data are consistent with the reported data, confirming that the product is HA.

### 3.2. Radiolabeling of ^32^P-labeled HA microparticles

In this study, the chemical synthesis (^32^P-Hap) and the physical adsorption (^32^P-doped-Hap) were used to prepare ^32^P-labeled HA to achieve maximum yield and excellent stability. The chemical synthesis exhibited higher radiolabeling yield and better stability than the common physical adsorption. NaH_2_PO_4_ and Na_2_H[^32^P]PO_4_ solutions were mixed to replace the NaH_2_PO_4_ in the synthesis of ^32^P-Hap. ^32^P was linked to the HA by covalent bonds, and thus the radiolabeling yield and stability were significantly improved. The yield of chemical synthesis (97.6 ± 0.5%) was higher than that of physical adsorption (92.7 ± 0.4%).

### 3.3. In vitro stability of ^32^P-labeled HA

**Figure 2.**
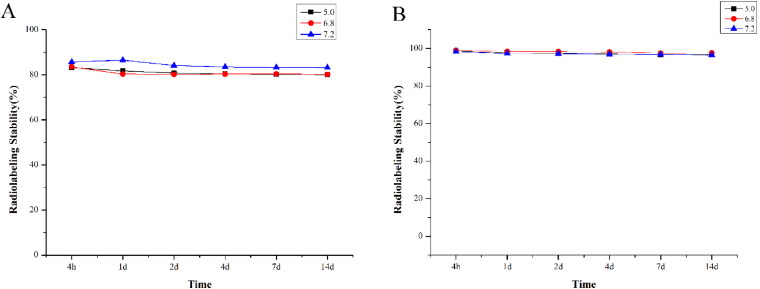
The in vitro stability at different pH of 5.0, 6.8, and 7.2 in saline, the radiochemical purity at each time interval of ^32^P-doped-Hap (A) and ^32^P-Hap (B).

The in vitro stability was investigated at different pHs of 5.0, 6.8, and 7.2 in saline. The results showed that pH had little effect on stability. ^32^P-Hap showed better stability than ^32^P-doped-Hap up to 14 days saline at room temperature. Figure 2(A) and 2(B) displays the radiochemical purity at each time interval of ^32^P-Hap and ^32^P-doped-Hap, respectively. After 14 days, the stability of ^32^P-Hap remained above 96% with a decrease of about 1.69%, while that of ^32^P-doped-Hap was only about 80% with a decrease of about 3.53%. The HA particles obtained by the physical adsorption were unstable and dissolved slowly.

### 3.4. In vivo antitumor efficacy

A 4T1 breast cancer mice model was established, and the antitumor efficacy of nano HA was started to be tested in vivo when the tumor volume reached approximately 200 mm^3^. We randomly divided the mice into 7 groups: saline, DOX, ^32^P-doped-Hap (100 μCi), ^32^P-Hap (50 μCi), ^32^P-Hap (100 μCi), ^32^P-Hap (150 μCi), and ^32^P-Hap (100 μCi) + DOX groups.

**Figure 3.**
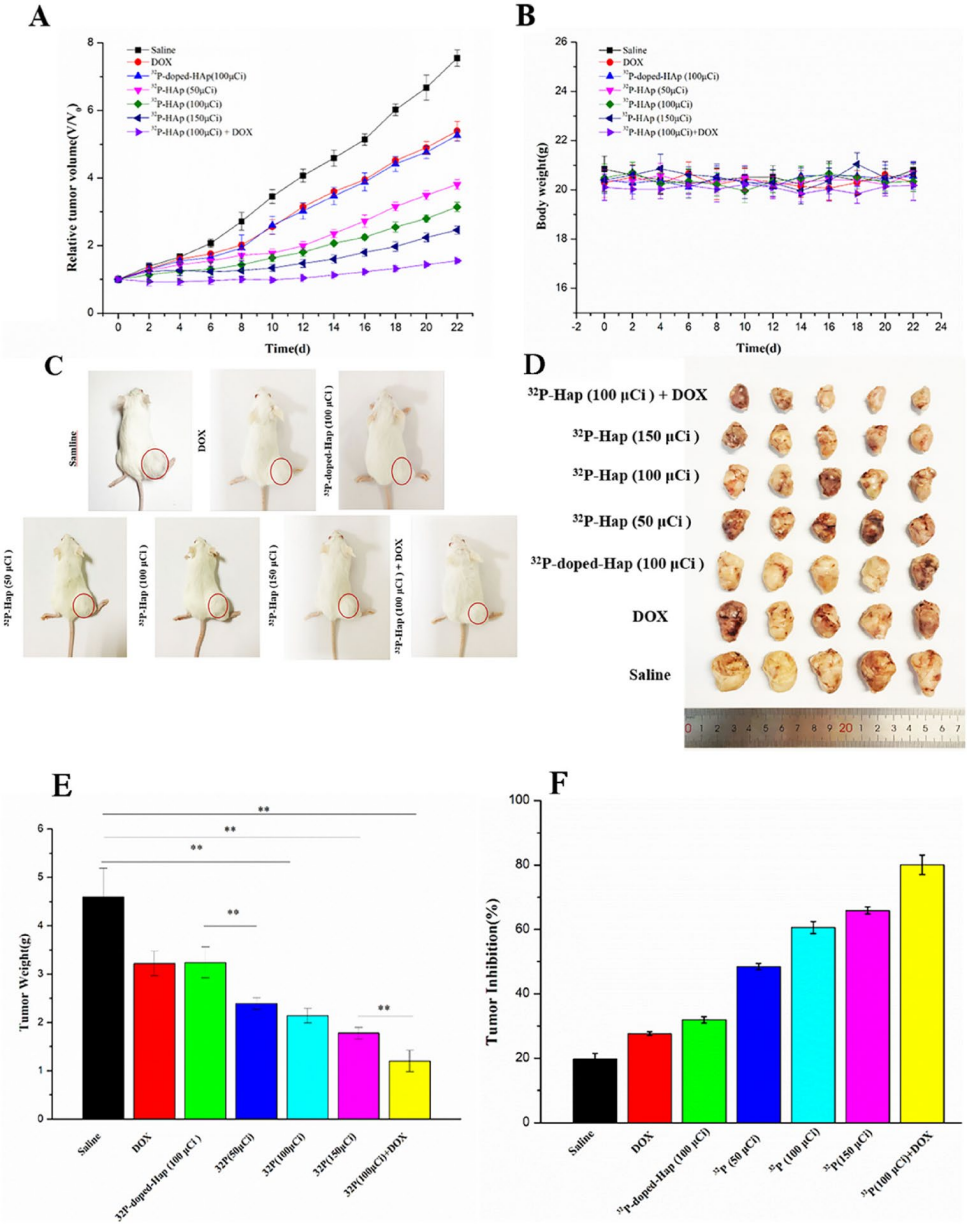
In vivo antitumor effect of ^32^P-Hap conjugates on bone tumor-bearing Balb/c model (female, 6–8 weeks old). Relative tumor volume (A) and body weight (B) and representative images of mice (C) and the xenograft tumors harvested form the mice (D) and weights of the harvested xenograft tumors (E) and tumor inhibition rate (F).

The volume of tumors within the specified time in each group is shown in Figure 3(A). The results indicated a low tumor inhibition rate of the DOX and ^32^P-doped-Hap (100 μCi) compared with saline. Furthermore, the effect of ^32^P-Hap (50 μCi) and ^32^P-Hap (100 μCi) on tumor volume reduction was not significant. In comparison, it was displayed that the tumor volume was decreased compared with the saline, DOX, and ^32^P-doped-Hap (100 μCi), due to their good tumor targeting capacity. The ^32^P-Hap (100 μCi) +DOX group exhibited the highest tumor suppressive effect and almost stopped the tumor progression. The body weight of mice was measured at various periods in the therapy. The results demonstrated no significant change in body weight between the saline and the ^32^P-Hap (100 μCi) +DOX groups, indicating that the mice were in a normal state during the treatment in Figure 3(B).

In addition, the tumors in the tibia of mice after treatments by different approaches and the representative images of tumors removed from the tibia of mice are shown in Figure 3(C) and 3(D), respectively. The ^32^P-Hap (100 μCi) +DOX group has the highest tumor suppression rate, possibly due to the combination of DOX with radiotherapy. The weight of tibial tumors in mice at the end of treatment is shown in Figure 3(E). Compared with the saline group, the tumor weight was significantly decreased in other groups, with the greatest decrease in ^32^P-Hap (100 μ Ci) + DOX group. Furthermore, tumor inhibition rates are shown in Figure 3(F) to evaluate the effect of antitumor after treatment. ^32^P-Hap (100 μCi) + DOX group exhibited the highest tumor inhibition rate (80.06%). The tumor inhibition rates in the ^32^P-Hap (100 μCi) (63.53%) and ^32^P-Hap (150 μCi) (69.93%) groups were higher than those in saline (17.36%) and DOX (26.99%) groups. Therefore, the growth of tumors can be significantly inhibited by 100 μCi ^32^P treatment, and tumor inhibition can be further enhanced by the DOX treatment.

**Figure 4.**
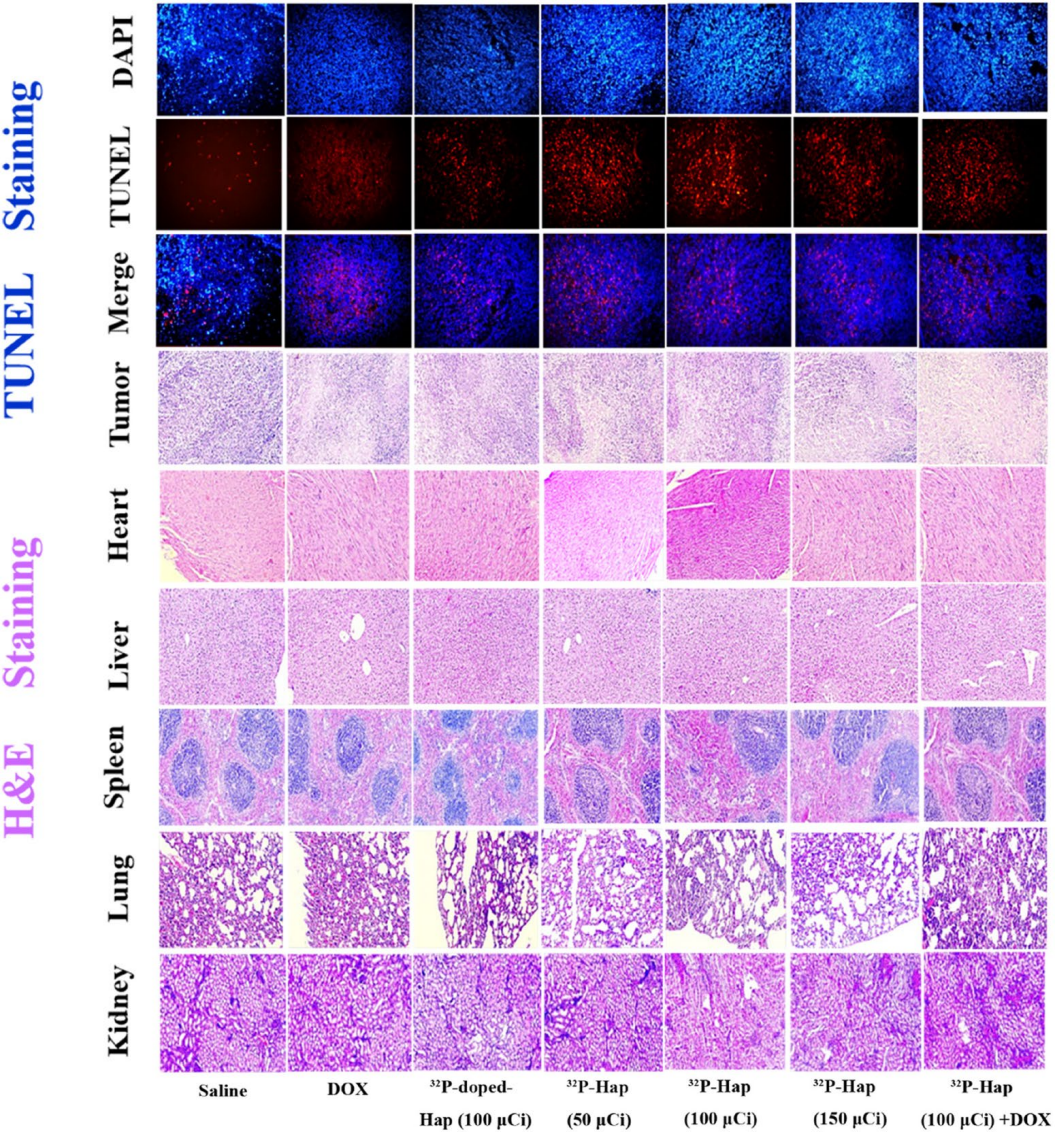
Representative images of TUNEL stained tumor slices, and H&E stained tumor, heart, liver, spleen, lung and kidney slices isolated from bone tumor-bearing Balb/c mice on day 21.

In vivo antitumor effects of various groups of nano-HA can be observed through TUNEL and H&E staining. The results are shown in Figure 4. The TUNEL results indicated that the number of apoptotic tumor cells in ^32^P-Hap (150 μCi), ^32^P-Hap (100 μCi) +DOX groups increased significantly compared with saline, DOX, ^32^P-doped-Hap (100 μCi), ^32^P-Hap (50 μCi), ^32^P-Hap (100 μCi), and ^32^P-Hap (150 μCi) groups. The apoptotic cells increased most after radio-chemotherapy. Furthermore, the tumor and each major organ were stained with H&E to evaluate the toxicity of each group after treatment. The results revealed that the staining of tumor sections displayed a similar regulation trend to TUNEL, and no lesioned tissue was found in the other groups. No local failure of cardiomyocytes, the morphologically intact hepatocytes without inflammatory cell accumulation, and no lesions in tissues (e.g. kidney congestion) were found.

### 3.5. Bone destruction assessment with CT

**Figure 5.**
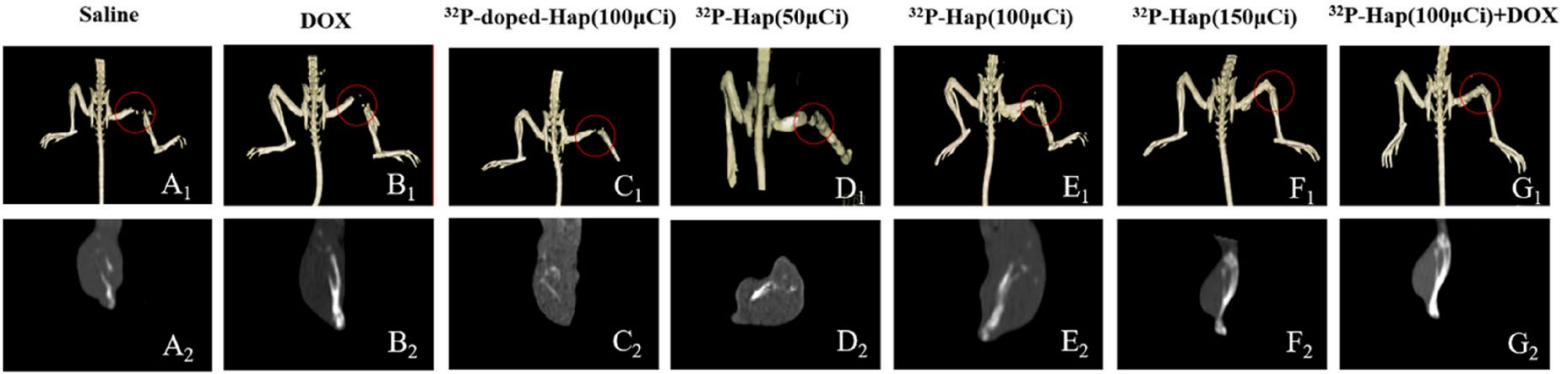
Three-dimensional reconstruction and sagittal section of images of the tibial tumor sites in mice with different treatments on day 21.

The incidence rate of malignant bone tumors is high, and bone tumors have a significant impact on the health of patients. The stimulated osteoclasts during the development of bone tumors lead to pathological bone destruction, which seriously affects the quality of life of patients. In this study, the skeletal morphology of tumor-bearing tibias after various treatments was further appraised using CT. As shown in Figure 5, the tumor-bearing site of mice leg tibia in Saline, DOX, groups are the most severely damaged owing to inefficient tumor suppression. However, the tumor suppression rates in ^32^P-doped-Hap (100 μCi), ^32^P-Hap (50 μCi), and ^32^P-Hap (100 μCi) groups are higher than those in saline and DOX groups. Furthermore, the structure of the mice tibia is intact in ^32^P-Hap (150 μCi) group, and partial corrosive lesions can still be observed. The complete structure of tumor-bearing tibias of mice in ^32^P-Hap (100 μCi) +DOX indicates that the treatment can effectively reduce bone resorption and efficiently protect the skeletal structure of bone from damage in bone tumors.

## 4. Discussion

Developing effective new drugs to cure cancer is a clinical challenge. In recent years, radiopharmaceuticals have become popular anticancer drugs. Most radionuclides are mainly used for to label materials. Nanomaterials have also attracted great attention in the biomedical field. HA is a biological material, and its chemical composition is similar to bone minerals. It has been applied widely in the medical field due to its excellent biocompatibility and bone tissue regeneration (Wijesinghe et al., 2014; Santos et al., 2017). These nanoparticles can be easily attached to osteosarcoma and osteoblast and can accelerate the growth of osteoblasts and the intake of osteosarcoma cells. In addition, the mammalian cells can be killed by the *β*-particles of radionuclide; ^131^I is preferred for thyroid carcinoma postoperative treatment (Pandi & Viswanathan, 2015), which has been used for decades; ^90^Y is marked of auxin to therapy the neuroendocrine tumor (Lorenzoni et al., 2018; Lawhn-Heath et al., 2021). ^32^P is suitable for treating tumors because of the long half-life nuclides.

The chemical synthesis has been proved as a promising candidate for synthesizing ^32^P-labeled HA particles with high radiochemical purity and stability in a simplified ^32^P labeling process. In addition, the ^32^P is a promising radionuclide for bone tumors (Yang et al., 2020), and DOX, also known as Adriamycin, is widely used to treat various cancers (Elsherbiny et al., 2016). Therefore, the ^32^P-Hap-DOX may be a potential drug for the targeted treatment of bone tumors. The radio-chemotherapy exhibits the most outstanding antitumor effect compared with other groups because the specific activity of radionuclide at the tumor site can emit radiation to kill tumor cells. Internal irradiation therapy has the shortest treatment time and the lowest risk of recurrence.

In this study, the ^32^P-labeled HA particles were obtained by physical adsorption and chemical synthesis, respectively, and a new labeling method for HA was constructed to treat bone metastases. Chemical synthesis has a higher radiolabeling rate and stability than physical adsorption. Additionally, ^32^P-Hap (100 μCi) + DOX has the strongest tumor suppression in vivo and effectively prevents bone destruction. This study demonstrates that chemical synthesis can improve the labeling efficiency of HA, and ^32^P-Hap (100 μCi) + DOX may be an effective strategy for bone metastasis treatments.

## 5. Conclusion

In summary, we have constructed a new labeling method for HA, which the goal of evaluation its utility as a promising candidate for the treatment of bone tumors has been successfully demonstrated. Our data demonstrated that it is possible to create stable nanosystems, which has a superior effect against bone cancer cells when compared with the physical synthesis, the radiolabeling rate and stability increased significantly. Also, the new labeling method can promote the development of radiopharmaceuticals.
