# Correction

**DOI:** 10.1080/10717544.2025.2610853

**Published:** 2026-01-21

**Article title:** Design and evaluation of ^32^P-labeled Hydroxyapatite nanoparticles for bone tumor therapy

**Authors:** Zhai, D., Wang, Y., Yu, S., Zhou, J., Song, J., Hao, S., & Chen, X.

**Journal:** *Drug Delivery*

**Bibliometrics:** Volume 30, Number 1, Pages 1−7

**DOI:** https://doi.org/10.1080/10717544.2023.2168791

The original version of our manuscript contained some inadvertent errors that presented in Figure 3C, and Figure 4.

Figure 3C and Figure 4 has been corrected, and the article is re-published.

